# Supplementary material for: Real-time observation of two distinctive non-thermalized hot electron dynamics at MXene/molecule interfaces
Source: Nat Commun. 2024 May 23;15:4406. doi: 10.1038/s41467-024-48842-9 (PMC11116487; doi:10.1038/s41467-024-48842-9)
Supplement: Supplementary file 1 — Supplementary Information [file 41467_2024_48842_MOESM1_ESM.pdf]

Supplementary Information for

Real-time observation of two distinctive non-thermalized hot electron dynamics at MXene/molecule interfaces

Qi Zhang[1,2], Wei Li[3,4], Ruixuan Zhao[1], Peizhe Tang[5], Jie Zhao[2], Guorong Wu[2], Xin Chen[3,4], Mingjun Hu[5], Kaijun Yuan[2,6,7*], Jiebo Li[1*], Xueming Yang[2,6,8]

1 Institute of Medical Photonics, Beijing Advanced Innovation Center for Biomedical Engineering, School of Biological Science and Medical Engineering, Beihang University, Beijing 100191, P.R. China.

2 State Key Laboratory of Molecular Reaction Dynamics and Dalian Coherent Light Source, Dalian Institute of Chemical Physics, Chinese Academy of Sciences, 457 Zhongshan Road, Dalian 116023, P.R. China.

3 Suzhou Laboratory, Suzhou 215123, Jiangsu, China.

4 GuSu Laboratory of Materials, Suzhou 215123 Jiangsu, China.

5 School of Materials Science and Engineering, Beihang University, Beijing 100191, P.R. China.

6Hefei National Laboratory, Hefei 230088, China.

7University of Chinese Academy of Sciences, Beijing 100049, China

8 Department of Chemistry and Center for Advanced Light Source Research, College of Science, Southern University of Science and Technology, Shenzhen 518055, China.

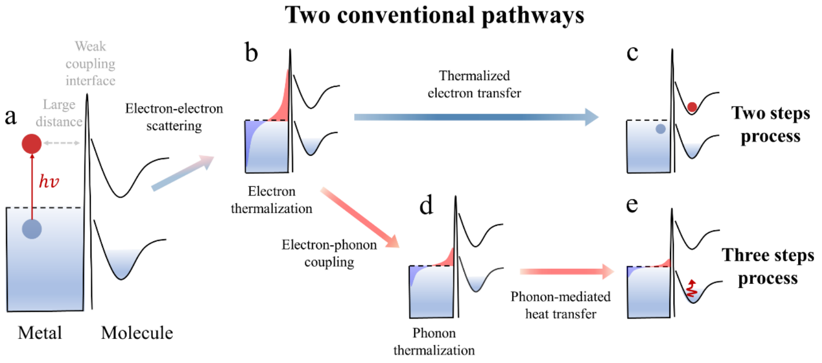

Supplementary Figure 1. The conventional electron and heat transfer pathways. Thermalized electron transfers (a-b-c, a two-step process): photo-induced non-thermalized hot electrons undergo electron-electron scattering and subsequently transfer to molecules; phonon-mediated heat transfer (a-b-d-e, a three-step process): the non-thermalized electrons undergo electron-electron scattering and electron-phonon coupling, followed by the thermalized phonon energy transfer into the adjacent surface molecules via interfacial phonon-phonon coupling. These two channels typically occur when non-thermal electrons are away from the interface or at a weak metal/molecule coupling interface. The region above the Fermi surface （dashed line） corresponds to the MXene conduction band, and the curves correspond to molecular potential energy surfaces. The connecting line between both elements corresponds to an energy barrier.

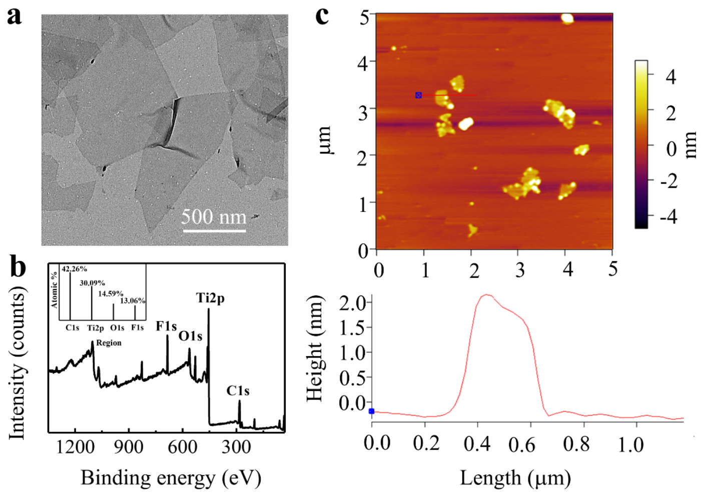

Supplementary Figure 2. Characterization of MXene. (**a**) TEM image. (**b**) XPS spectrum. (**c**) AFM image of $Ti_3C_2T_x$ on silicon (upper panel) and a corresponding height of the flake (lower panel).

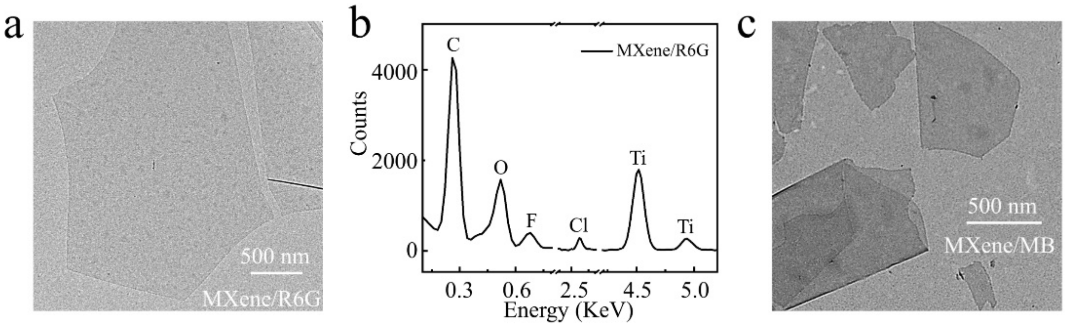

Supplementary Figure 3 (**a**) TEM image of MXene/R6G. (**b**) Energy dispersive spectroscopy (EDS) result of the MXene/R6G sample. The element of Cl comes from R6G. (c) TEM image of MXene/MB.

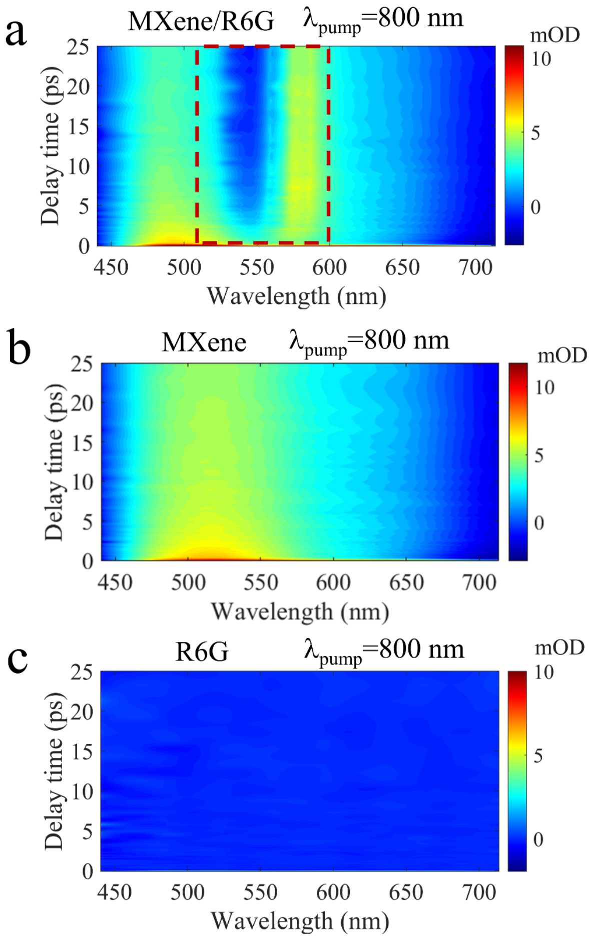

Supplementary Figure 4. **Two-dimensional plot of TA spectra with excitation of 800 nm.** (**a**) MXene/ R6G film. The region within the red square frame shows the signal from R6G. (**b**) MXene film. (**c**) R6G film. To rule out the possibility of direct excitation of the molecule, Fig. **c** exhibited no TA signal above the noise level, suggesting that direct excitation of the molecule cannot occur.

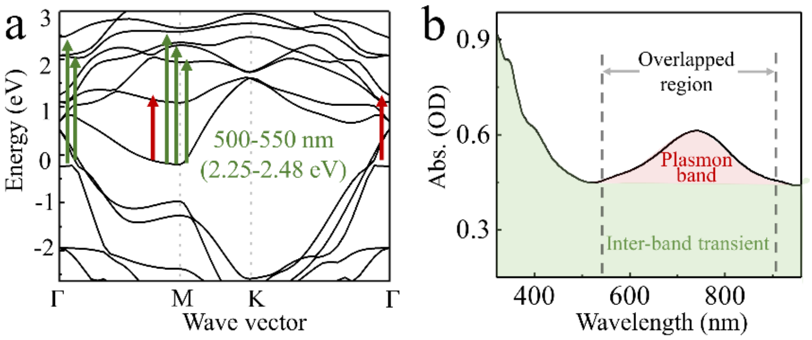

Supplementary Figure 5. (**a**)The calculated band structures of $Ti_3C_2O_2$ MXene. The green arrow represents the possible inter-band transitions of electrons in MXene after absorption of visible light. While the red arrow represents near-infrared light absorption (b) A steady-state absorption spectrum of MXene.

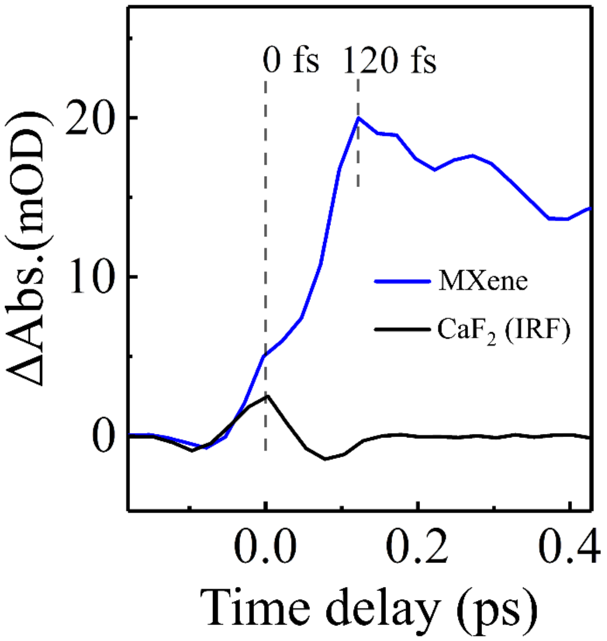

Supplementary Figure 6. Measuring time-zero in CaF_2 and MXene's electron-electron scattering process. The blue line represents measuring MXene dynamics, while the black line represents the transparency of CaF_2 dynamics probed at 510 nm after excitation at 800 nm. The time zero can be obtained by generating off-resonantly coherent artificial signals in a CaF_2 substrate after excitation. The MXene sample supported by CaF_2 allowed us to observe a small artificial signal, which enabled us to accurately determine the time zero. This process corresponds to electron-electron scattering, and its duration is approximately 120 fs.

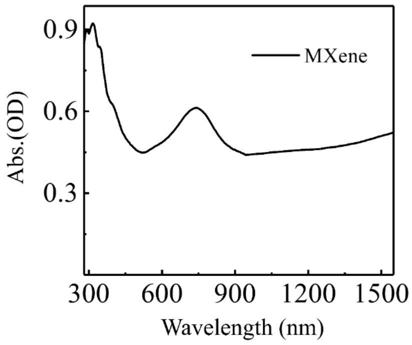

Supplementary Figure 7. The ultraviolet-visible and near-infrared absorption spectrum of MXene film.

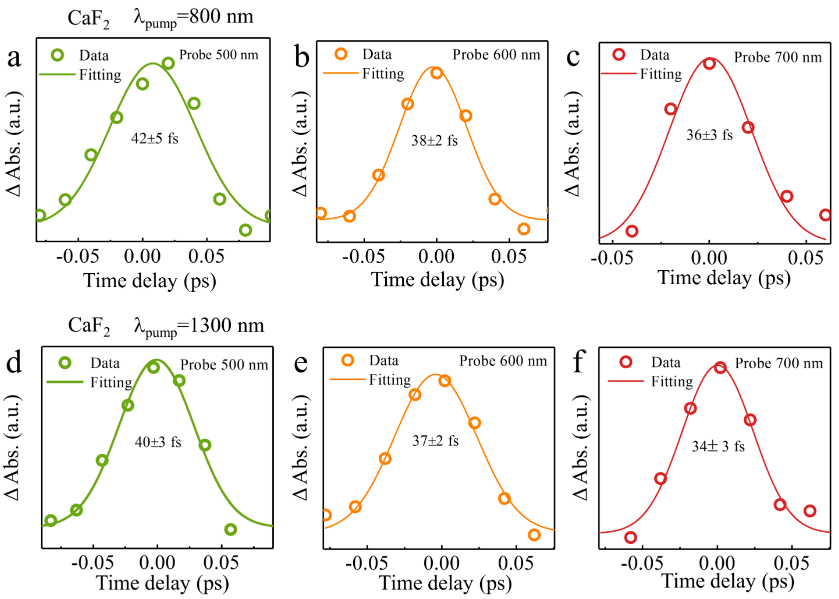

Supplementary Figure 8. Measuring IRF with CaF_2 of thickness of 1 mm probed at various wavelengths with two pump pulses (a-c) 800 nm. **(d-f)** 1300 nm. The circles are data and the lines are fitting results with a Gaussian function

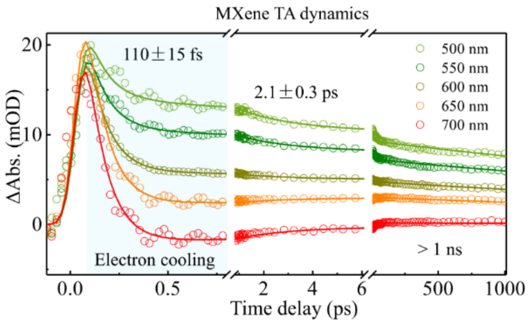

Supplementary Figure 9. TA dynamics traces of MXene probed at different wavelengths under the excitation at 800 nm and the corresponding fitting results of MXene.

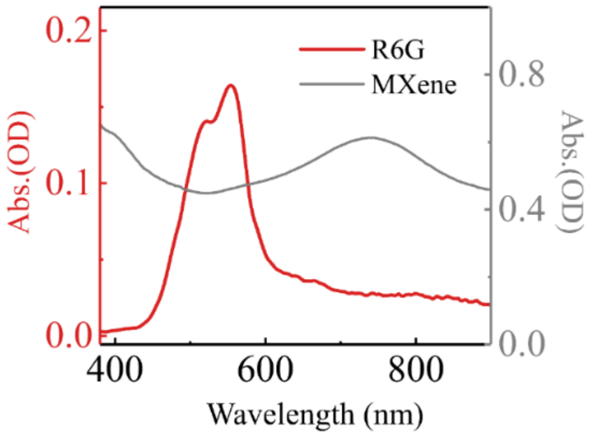

Supplementary Figure 10. Steady-state absorption spectra of the MXene film (grey) and isolated R6G film (red).

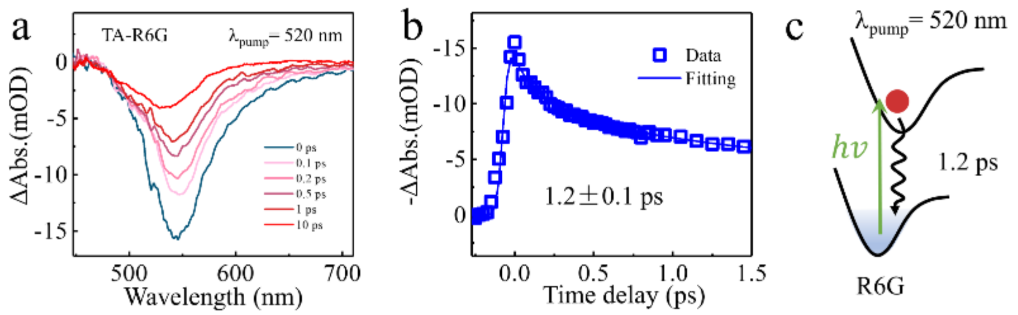

Supplementary Figure 11. (**a** & **b**) TA spectra and dynamic at 545 nm in isolated R6G film after 520 nm excitation. (**c**) Schematic of an excited electron in isolated R6G film after 520 nm excitation.

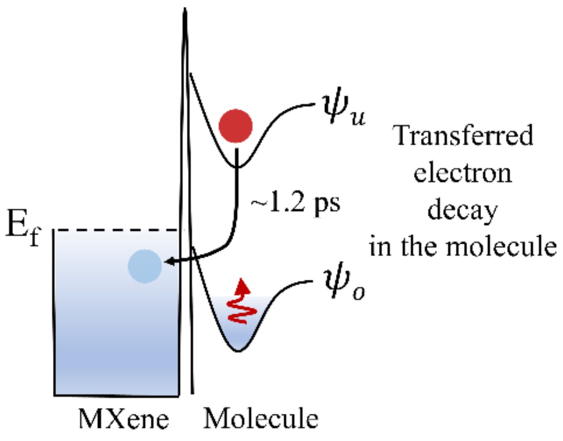

Supplementary Figure 12. Schematic of transferred electron decay in molecules and then returns to MXene.

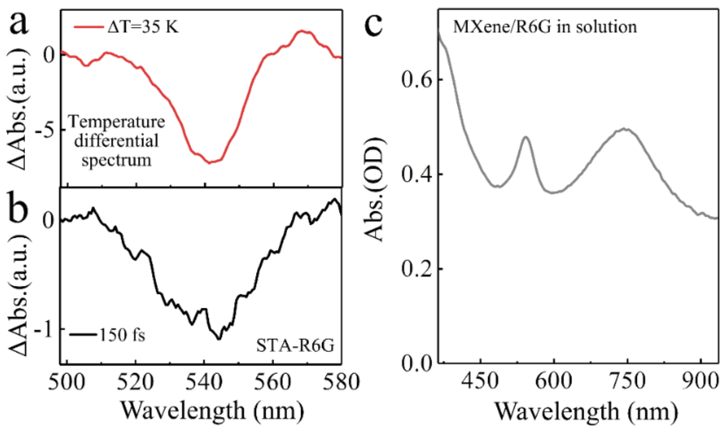

Supplementary Figure 13. Comparison between a temperature differential and a STA spectrum of MXene/R6G in aqueous solution: (**a**) A temperature differential spectrum (obtained by subtracting a steady-state absorption spectrum at high temperature from one at low temperature). (**b**) An STA-R6G spectrum at 150 fs after excitation at 800 nm. (**c**) A steady-state absorption spectrum of MXene/R6G in aqueous solution.

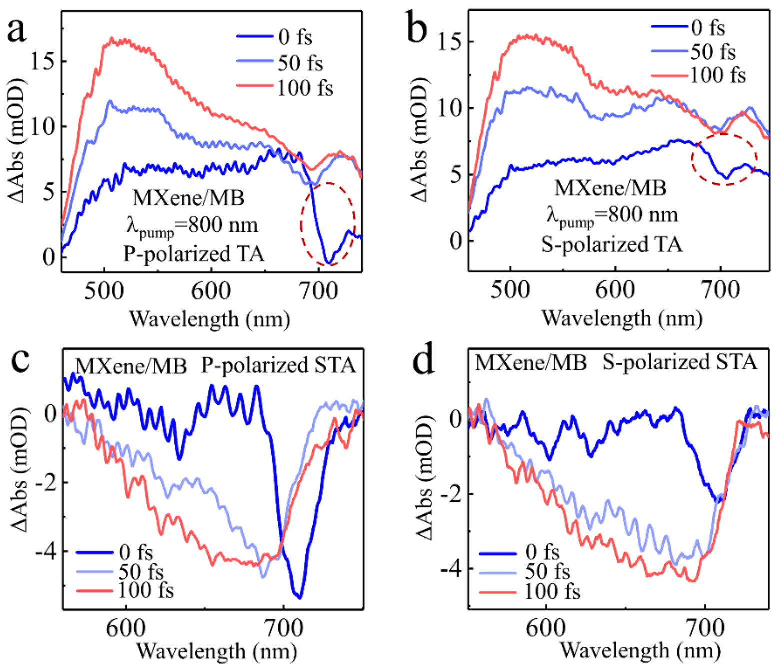

Supplementary Figure 14. Polarization-dependent TA and STA spectra in MXene/MB. (**a&b**) P- and S-polarized TA. (**c&d**) P- and S-polarized STA. The signal in the MB's regime (indicated by the red circle) is stronger with P-polarization in Fig. **a** compared to the signal with S-polarization in Fig. **b**

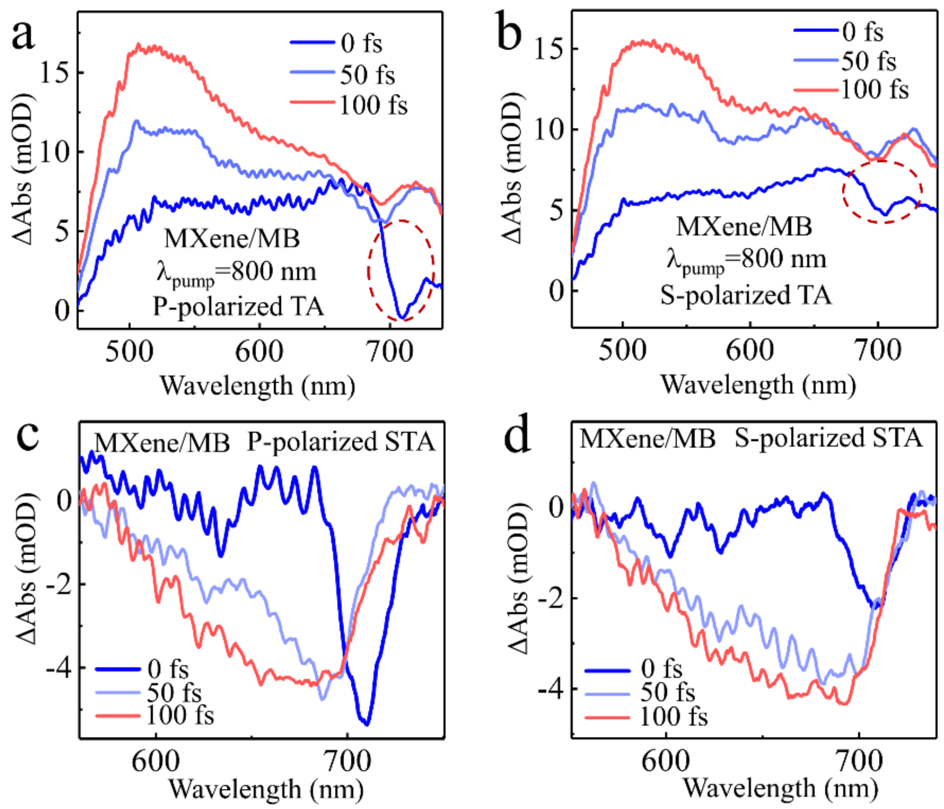

Supplementary Figure 15. Polarization-dependent TA and STA spectra in MXene/R6G. (**a&b**) P- and S-polarized TA. The dashed circled marks indicate significant differences in signal intensities between P and S-polarized TA. (**c&d**) P- and S-polarized STA.

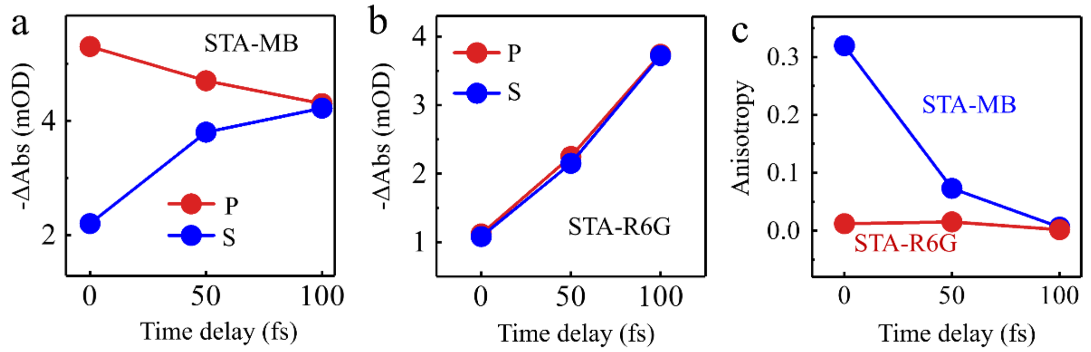

Supplementary Figure 16. (**a&b**) Polarization-dependent STA-MB and STA-R6G dynamics were probed at peak intensity for each delay time. (**c**) Calculated anisotropy (R) as a function of time delay for STA-MB and -R6G. The polarization dependence of spectral signals can be quantified by anisotropy (R), defined as R = (P-S) / (P+2S).

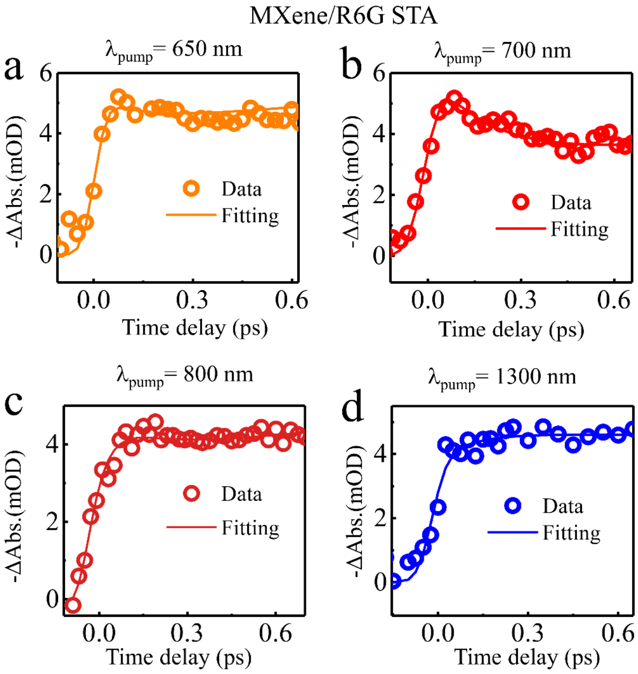

Supplementary Figure 17. The dynamics of STA probed at peak position with various pump wavelengths in MXene/R6G. (**a**) pump at 650 nm (**b**) pump at 700 nm (**c**) pump at 800 nm (**d**) pump at 1300 nm.

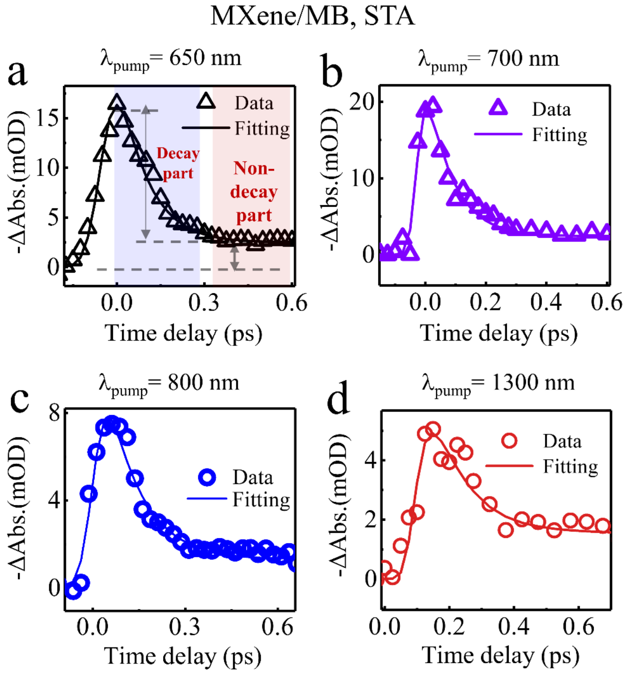

Supplementary Figure 18. The dynamics of STA probed at peak position with various pump-wavelengths in MXene/MB. (**a**) pump at 650 nm (**b**) pump at 700 nm (**c**) pump at 800 nm (**d**) pump at 1300 nm.

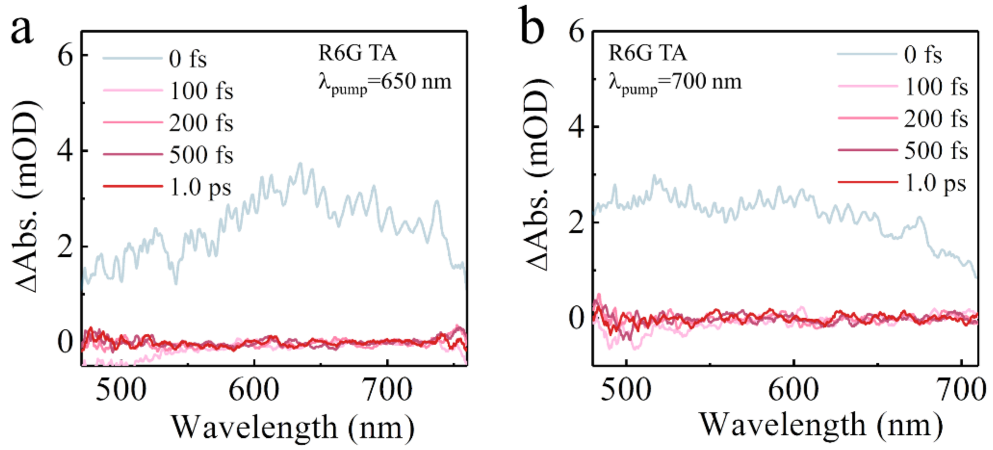

Supplementary Figure 19. The TA spectra of isolated R6G film. (**a**) Pump at 650 nm. (**b**) Pump at 700 nm. The signal at 0 fs is artificial owing to an off-resonantly generated coherent artifact from a substrate (CaF_2).

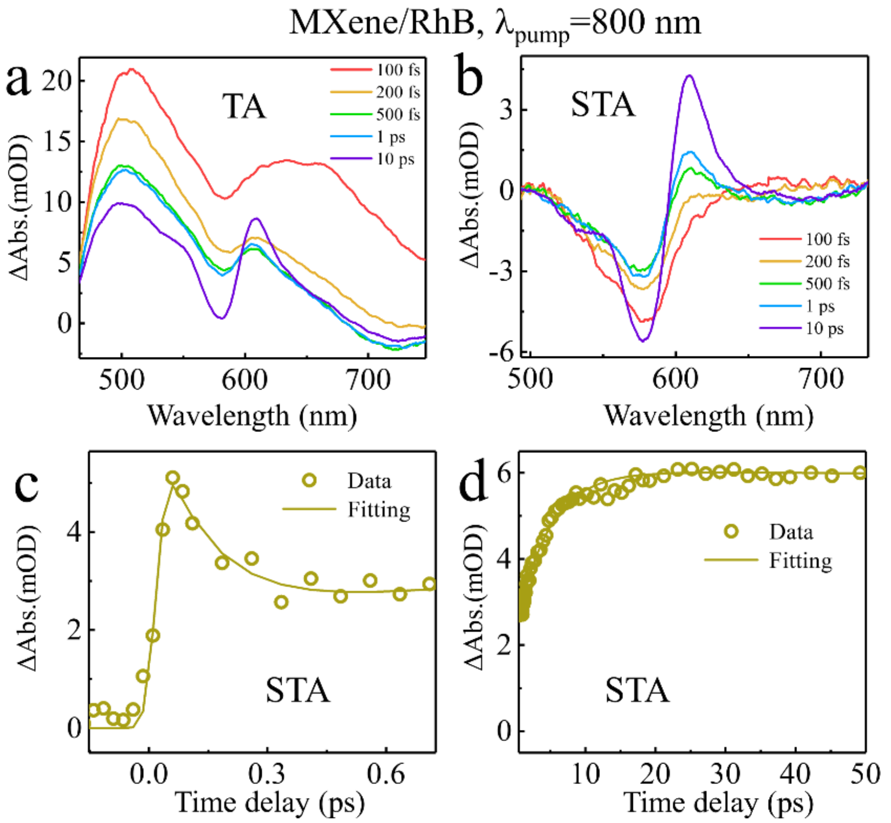

Supplementary Figure 20. Transient spectra and dynamics of MXene/RhB after 800 nm excitation. (**a&b**) TA and STA spectra, respectively, at indicated delay time **(c&d)** Early and later dynamics at an indicted time interval, respectively.

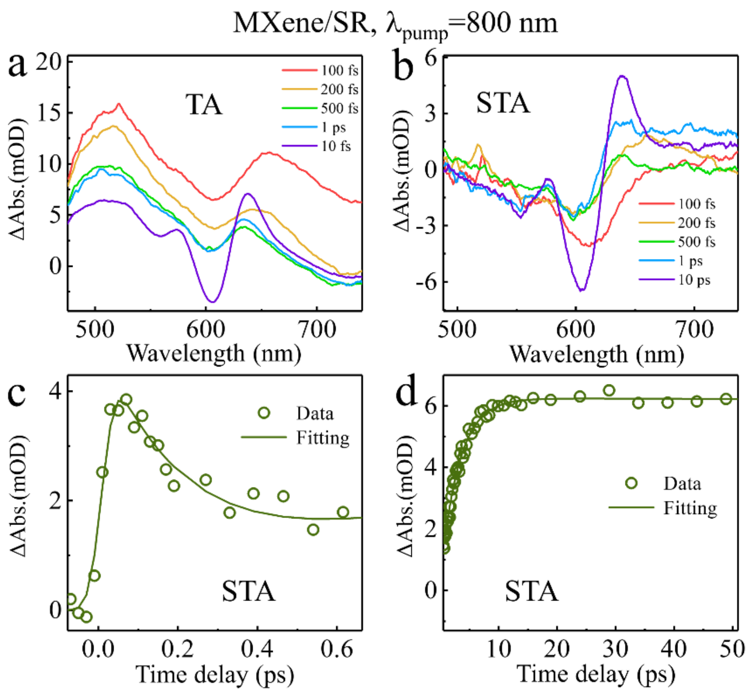

Supplementary Figure 21. Transient spectra and dynamics of MXene/SR after 800 nm excitation. (**a&b**) TA and STA spectra, respectively, at indicated delay time **(c&d)** Early and later dynamics at an indicted time interval, respectively.

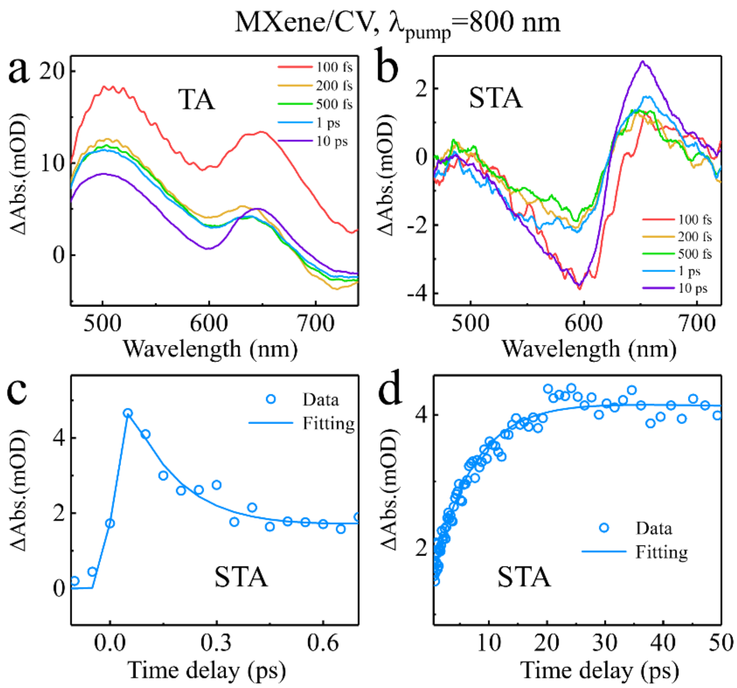

Supplementary Figure 22. Transient spectra and dynamics of MXene/CV after 800 nm excitation. (**a&b**) TA and STA spectra, respectively, at indicated delay time **(c&d)** Early and later dynamics at an indicted time interval, respectively.

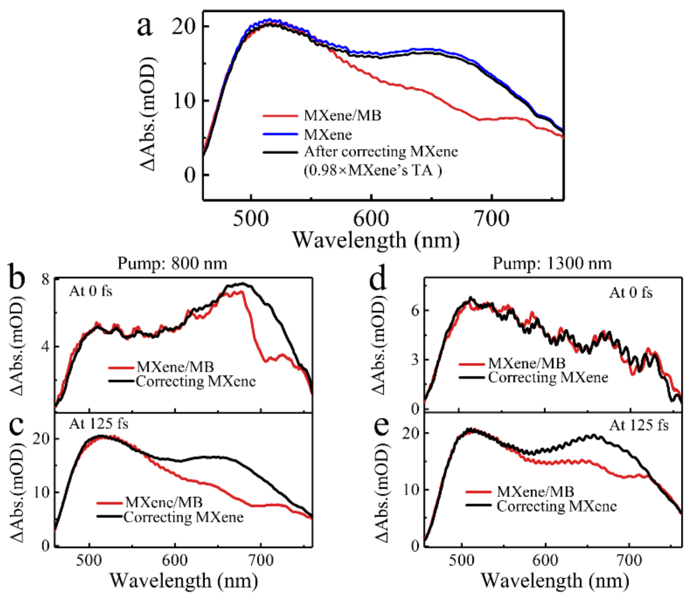

Supplementary Figure 23. Processing MXene spectral and spectral comparison between MXene/MB (red lines) and adjusted MXene (black lines) at selected time. **(a)** Showing the MXene's TA (blue line), after correcting MXene's TA (black lines) and original MXene/MB's TA spectra. **(b&c)** Pump 800 nm at 0 fs and 125 fs, respectively. (**d&e**) Pump 1300 nm at 0 fs and 125 fs, respectively.

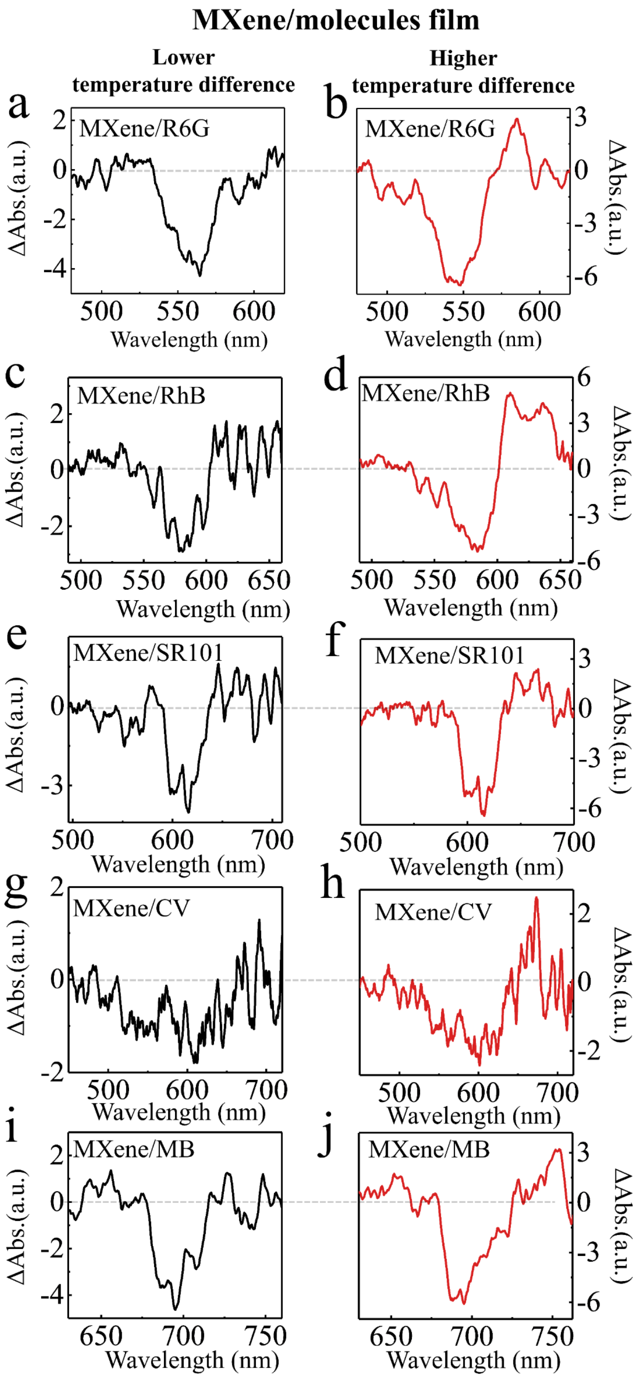

Supplementary Figure 24. High- and low-temperature difference (ΔT <40 K and ΔT>40 K) spectra of MXene/molecules film. (**a&b**) MXene/R6G film. (**c&d**) MXene/RhB film. (**e&f**) MXene/SR101 film. (**g&h**) MXene/CV film. (**i&j**) MXene/MB film. These pure molecular temperature differential absorption spectra in MXene/molecules are obtained by subtracting the temperature differential absorption spectra of MXene/molecule (Supplementary Note 2). The a.u. in the y-axis label expresses arbitrary unit.

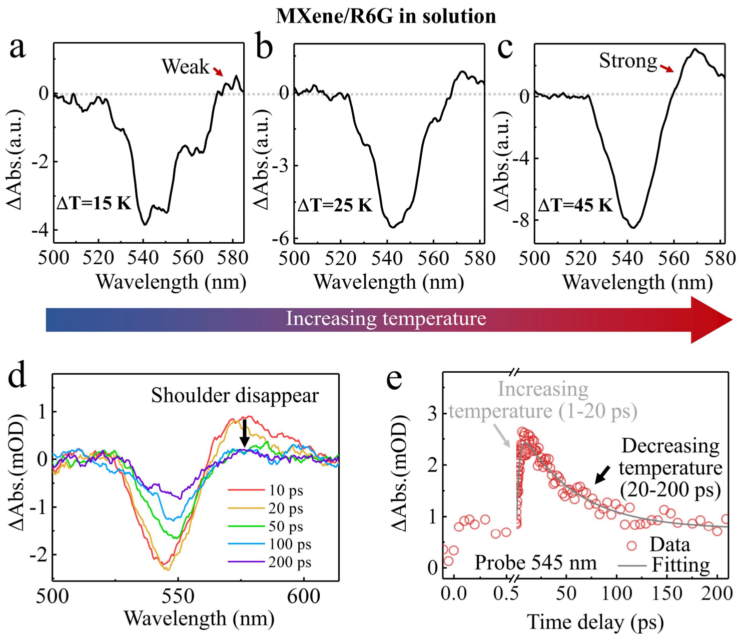

Supplementary Figure 25. MXene/R6G in aqueous solution. **(a-c)** Various temperature differential (ΔT=15, 25, 45 K) absorption spectra. The reference temperature is 295 K (room temperature). The a.u. in the y-axis label expresses arbitrary unit. (**d**) STA-R6G spectra. (**e**) STA-R6G dynamics probed at 545 nm.

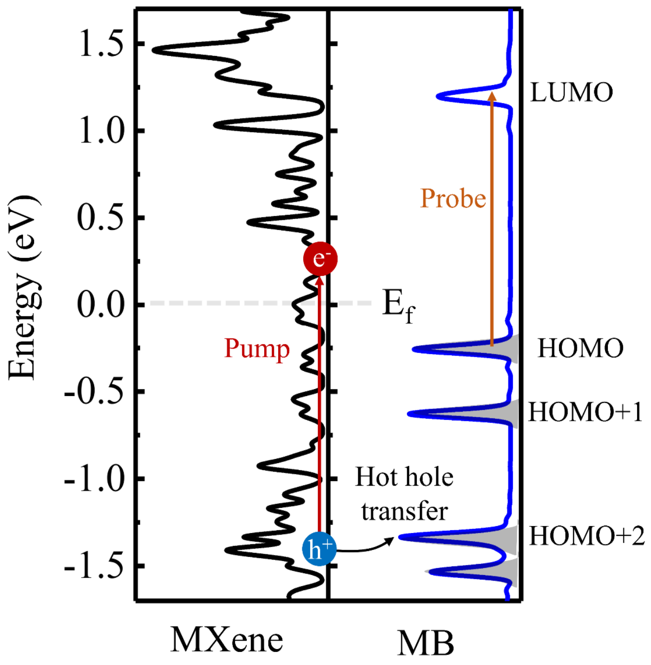

Supplementary Figure 26. Partial density of states of entire MXene/MB. The red arrows represent a photo-induced generation of hot holes, and the orange arrows indicate the probing wavelength.

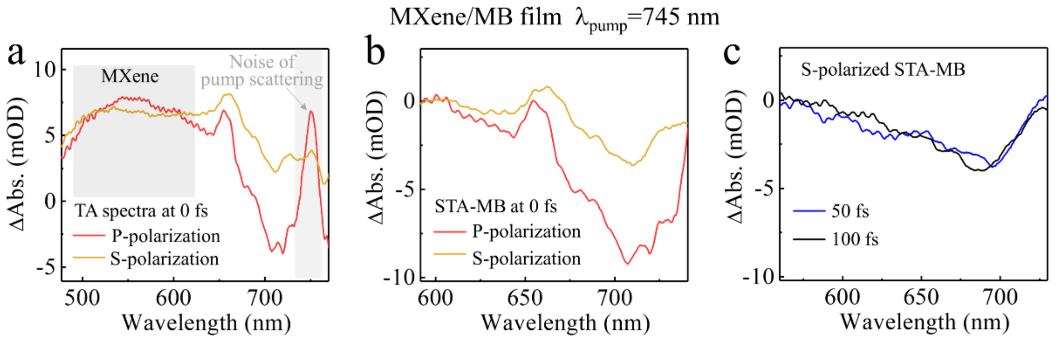

Supplementary Figure 27. Polarization-dependent TA and STA spectra in MXene/MB with excitation at 745 nm. (**a**) P- and S-polarized TA at 0 fs. (**b**) P- and S-polarized STA at 0 fs. (**c**) S-polarized STA at 50 and 100 fs. The selected excitation at 745 nm rather than at 700 nm was caused by the strong noise of pump scattering, which hindered us from obtaining accurate data ranging from 680-720 nm.

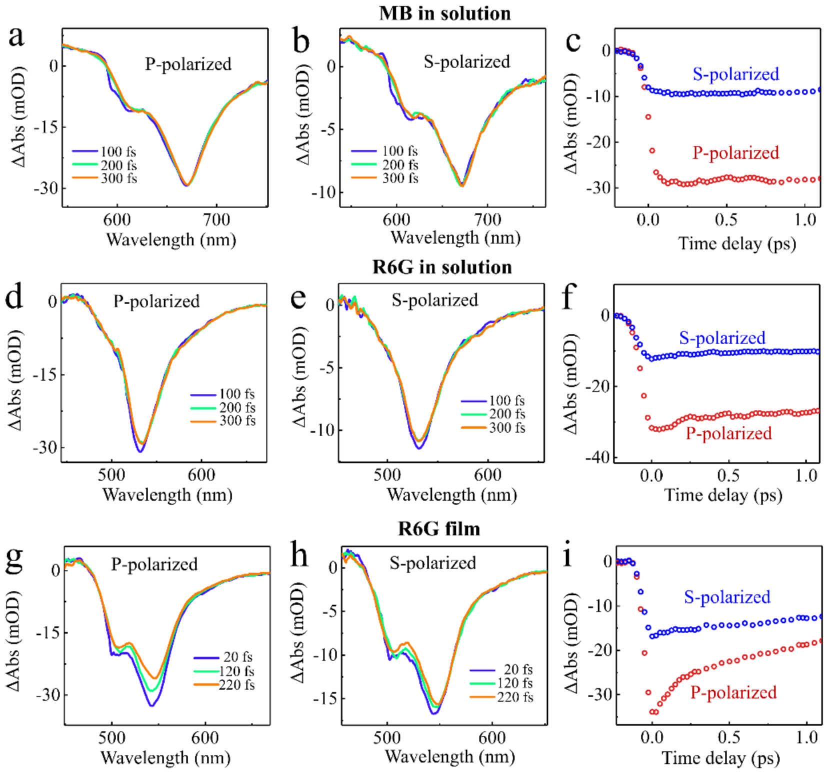

Supplementary Figure 28. Polarization-dependent transient spectra and dynamics of MB and R6G in aqueous solution and R6G film. (a-c) MB in aqueous solution after 620 nm excitation, P- and S-polarized dynamics probed at 670 nm. (d-f) R6G in aqueous solution after 510 nm excitation, P- and S-polarized dynamics probed at 530 nm. (g-i) R6G film with excitation at 510 nm, P- and S-polarized dynamics probed at 530 nm.

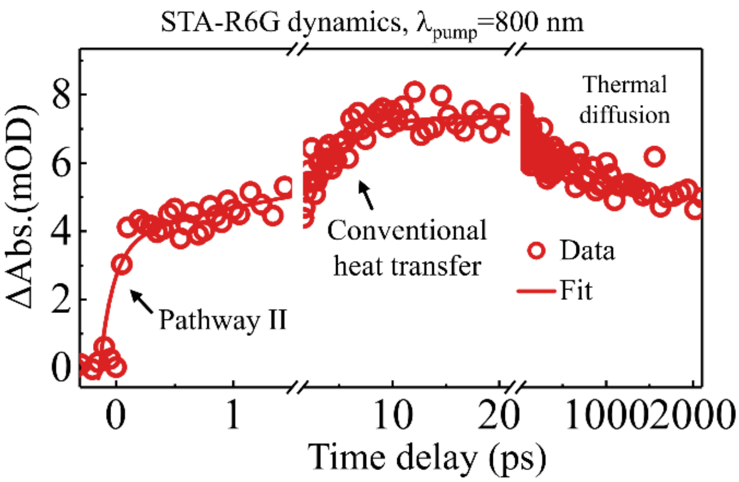

Supplementary Figure 29. Dynamics of the STA MXene/R6G data (dots) and its fit (line) probed at 545 nm after 800 nm excitation. Between 0 to 200 fs, representing the first rise of heating the molecule by the non-thermalized electron interfacial scattering (pathway II). The second rise is between 1 and 15 ps, representing the Conventional heat transfer (PMHT) by vibration-vibration coupling. The decay process between 20 ps and 2000 ps represents the heat diffusion to the surroundings.

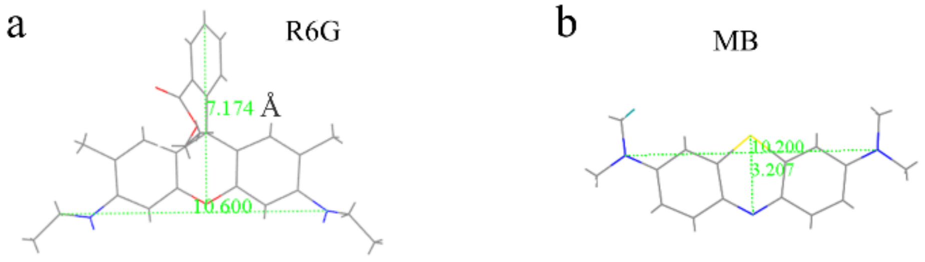

Supplementary Figure 30. Calculated (**a**) MB and (**b**) R6G molecular structures and measured size.

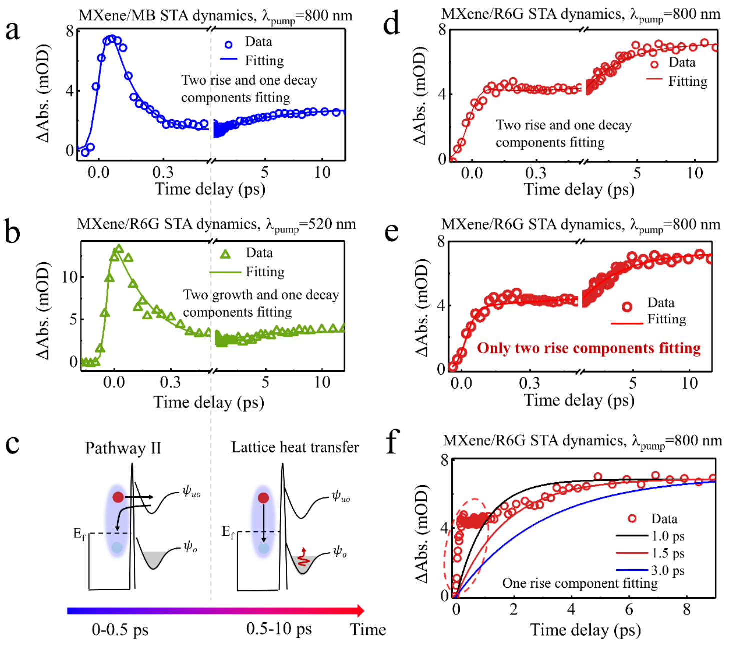

Supplementary Figure 31. Fitting analysis electron/heat transfer pathways. (a) STA dynamics and fitting results in MXene/MB after 800 nm excitation. (**b**) STA dynamics and fitting results in MXene/R6G after 520 nm excitation. (**c**) Schematic illustrating two mechanisms of excited electron relaxation at different time intervals. (**d-f**) STA dynamics and various fitting analyses in MXene/R6G after 800 nm excitation. Two growth and one decay components are used for data fitting in Fig. **d**. In Fig. **e**, only two growth components are utilized, showing that the data is consistent with a two-exponential growth process. Finally, Fig. **f** employs a single growth component for data fitting, revealing that the data does not align with a one-exponential growth process.

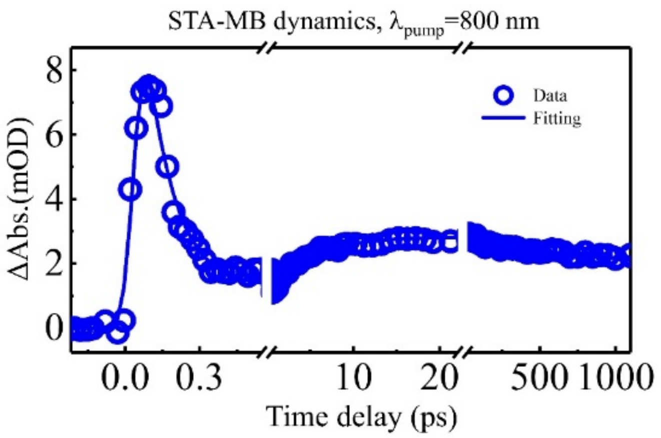

Supplementary Figure 32. The STA-MB dynamics probed at 680 nm after excitation of 800 nm.

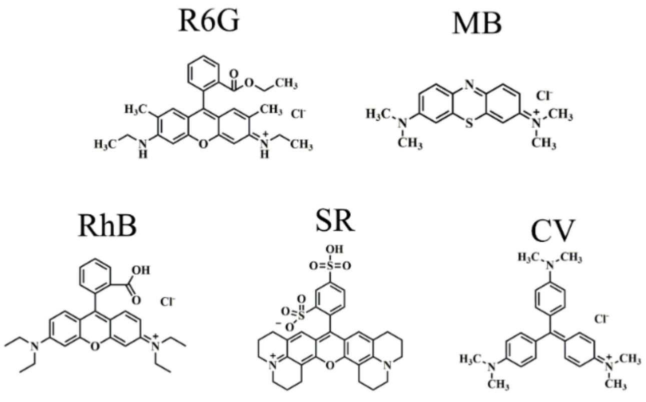

Supplementary Figure 33. Five molecular structures.

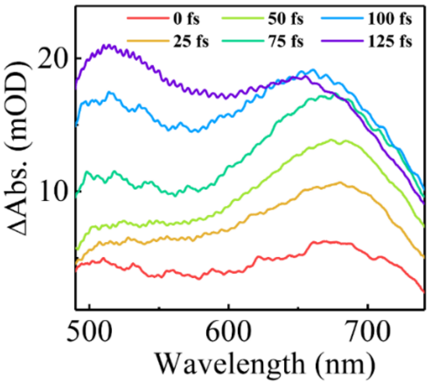

Supplementary Figure 34. TA spectra of MXene film after excitation of 800 nm within 125 fs.

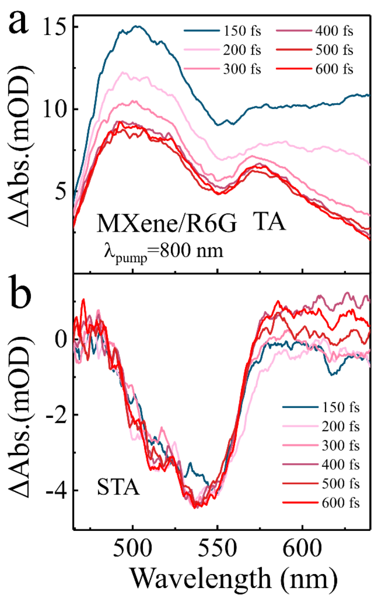

Supplementary Figure 35. The spectra of MXene/R6G during 150-600 fs after excitation of 800 nm. (**a**) The TA spectra. (**b**) The STA spectra.

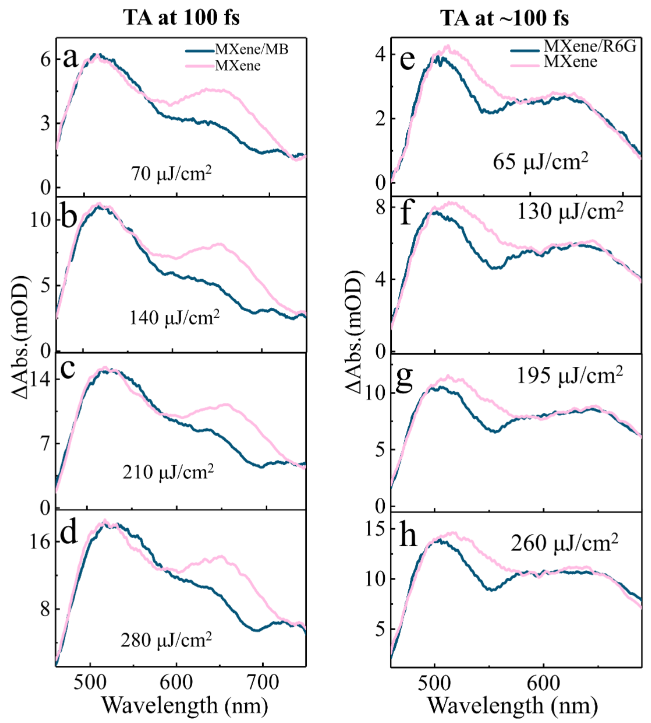

Supplementary Figure 36. The TA spectra of MXene/molecules and MXene under different pump fluences after 800 nm excitation probe at 100 fs. (**a-d**) MXene/MB. (**e-h**) MXene/R6G.

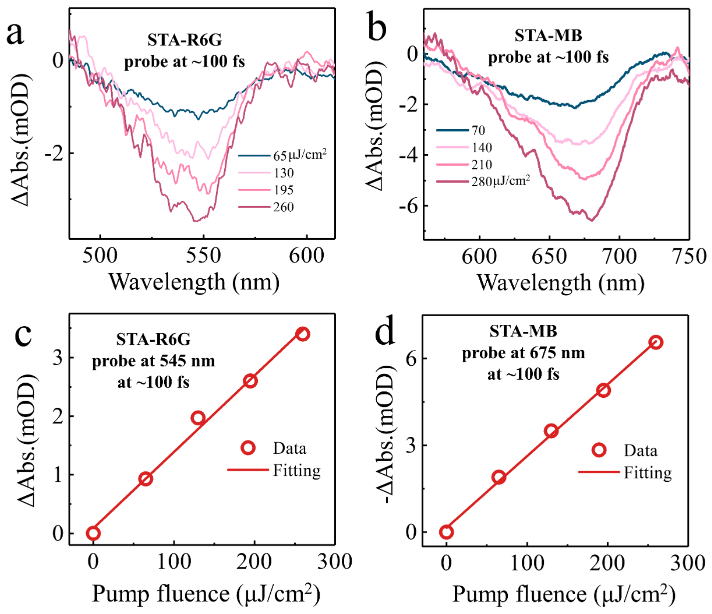

Supplementary Figure 37. The STA spectra and corresponding signal sizes under different pump fluences after 800 nm excitation. (**a&b**) STA-MB and STA-R6G spectra at 100 fs at indicated pump fluences. (**c&d**) STA-MB and R6G signal sizes probed at peak position as a function of pump fluences at 100 fs, respectively.

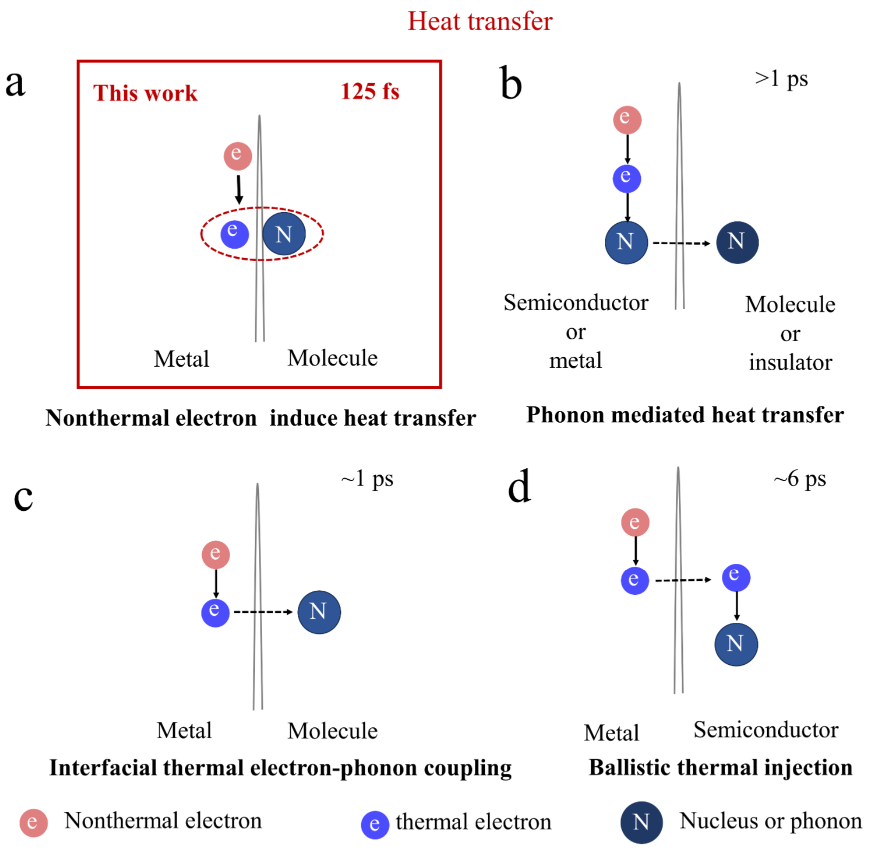

Supplementary Figure 38. Detailed comparisons of these interfacial heat transfer. (**a**) A new mechanism was discovered in this work, for heat transfer across a MXene/molecule interface. Non-thermalized electron heat transfer via scattering with an interfacial electron and nucleus coupling state that is localized in the MXene and molecule across the interface. During this non-thermalized electron relaxation with the interfacial coupling state, both the molecular nucleus and MXene's electrons are heated simultaneously. (**b**) Non-thermalized electrons heat the metal nucleus after experiencing electron-electron scattering and electron-phonon coupling in the semiconductor, then the molecular nuclei are heated by a hot semiconductor lattice[1]. This mechanism is applicable in metal/insulator interface[2]. (**c**) In some metal/molecule interfaces, generated thermalized electron coupling with molecular nuclei after electron-electron scattering[3]. (**d**) In some metal/semiconductor interfaces, thermalized electrons in metal transfer energy to electrons in the semiconductor, and then the electrons in the semiconductor couple to its phonons[4]. The dashed arrow represents energy transfer without any concomitant charge flow.

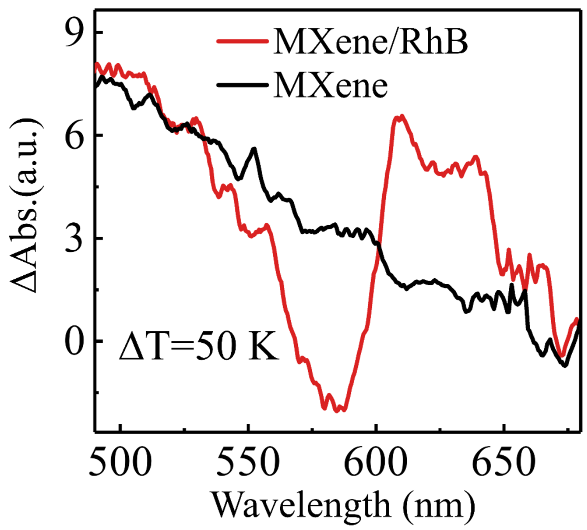

Supplementary Figure 39. The red line displays a differential absorption spectrum of MXene/RhB without subtracting a differential absorption spectrum of MXene, and the black line displays a differential absorption spectrum of MXene.

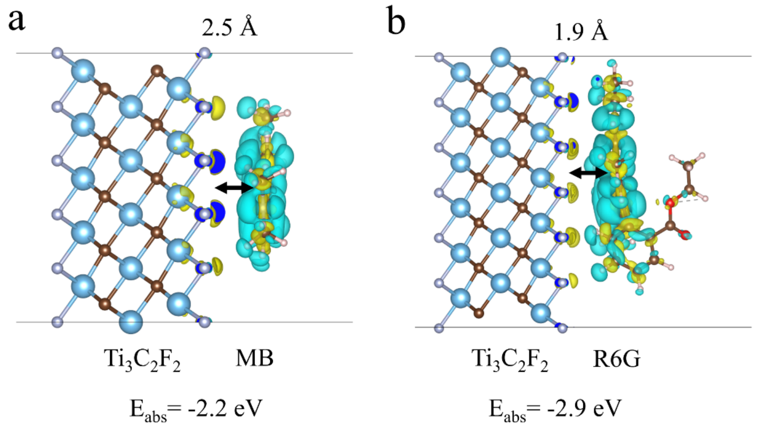

Supplementary Figure 40. Calculated charge density differences of fluorine-terminated MXene/molecules. (**a**)$Ti_3C_2F_2$/MB. (**b**) $Ti_3C_2F_2$/R6G. Compared to $Ti_3C_2O_2$/MB, there are some changes in charge distribution, adsorption energy, and interface distance. Hence, the surface terminations may change the electron/heat transfer rate and efficiency.

Supplementary Table 1. List of the peak position of absorbing photonic wavelength/energy of MXene and Molecules complexed with MXene, according to Fig. 2b.

Species	Peak position (nm)	Peak position absorbing photo energy (eV)
MXene	740	1.68
R6G	545	2.27
RhB	580	2.12
SR	605	2.05
CV	605	2.05
MB	685	1.81

Supplementary Table 2. List of the peak position differences of absorbing photonic wavelength/energy of MXene and Molecules complexed with MXene, according to Fig. 2b.

Species	Peak position difference between MXene and molecules (nm)	Energy difference between MXene and molecules (eV)
MXene/R6G	195	0.59
MXene/RhB	158	0.44
MXene/SR	134	0.37
MXene/CV	134	0.37
MXene/MB	55	0.13

Supplementary Note 1. The area ratio of molecules covered to $Ti_3C_2T_x$.

The area ratio of molecules covering $Ti_3C_2T_x$ can be calculated based on the molecular area and the area of MXene within a predetermined concentration of the mixed solution. The area of MXene can be calculated using the following formula:

$$s_M = \frac{m_M}{\rho_M * h_M}$$

For MXene, S_M represents the surface area, h_M is the thickness (2 nm), and ρ_M is the density (3.19 g/cm^3)[5]. In a 1 mL solution, the surface area of 0.1 mg of MXene is calculated to be 1.6×10^{-2} m^2. The surface areas of R6G and MB are approximately 0.38 nm^2 and 0.33 nm^2, respectively, based on their calculated molecular size as shown in Supplementary Fig. 30. The concentrations of these molecules are around 1.0×10^{-5} mol/L. Consequently, the 1 mL solution contains approximately 9.03×10^{15} molecules. For R6G and MB in a 1 mL aqueous solution, their surface areas are estimated to be 2.27×10^{-3} m^2 and 1.99×10^{-3} m^2, respectively. As a result, the area ratios of molecules covering the $Ti_3C_2T_x$ surface range from 12% to 14%. The low molecular concentration is intentional to prevent aggregation, as higher concentrations could adversely affect the dynamics and transient absorption spectra in the MXene/molecule system, making it challenging to obtain accurate STA spectra.

Supplementary Note 2. The steady-state absorption and temperature differential spectra.

A UV-Visible-Near Infrared light source, comprising a deuterium and halogen lamp, was employed to record the steady-state absorption spectra. The light emitted by the source passed through the samples before being focused onto a fiber using a convex lens. Spectral signals were captured by a fiber spectrometer, either AvaSpec-ULS2048CLEVO or AvaSpec-NIR256-1.7-HSC-EVO. The absorption spectra were determined by calculating the changes in absorbance between the samples and the background. For the measurement of absorption spectra at different temperatures, a temperature controller (Transmit G9) was integrated into the setup. The samples were dispersed in an aqueous solution within a cuvette with a thickness of 2 mm (Starna Scientific, Type 21/Q/2). The temperature of the samples was monitored and maintained by the temperature controller. After each target temperature was reached, a

stabilization period of 10 minutes was allowed. Subsequently, the temperature differential spectra were generated by calculating the change in absorption between the high-temperature absorption spectra and the room-temperature absorption spectra. For MXene/molecule, the pure molecular temperature differences absorption spectra are obtained by subtracting the temperature differences absorption spectra of MXene/molecule (red line in Supplementary Fig. 39) from the MXene (black line in Supplementary Fig. 39). To demonstrate universality, the observation of STA spectra may be caused by heated molecules, we have performed the differential absorption spectra of all the investigated MXene/molecules as shown in Supplementary Figs. 24 and 25. The differential absorption spectra at low-temperature differences ($\Delta T<40$ K) exhibit only negative peaks. While they show both positive and negative peaks at high-temperature differences ($\Delta T>40$ K). The results are almost consistent with STA spectra (Supplementary Figs. 20-22).

Supplementary Note 3. Calculated the non-decay and decay part of STA dynamics.

The STA dynamics, probed at the peak position for different MXene/molecules after various pump-wavelength excitations, are presented in Supplementary Figs. 17-18 and 20-22c. The summarized non-decay components are presented in Fig. 5e and f. The population of non-decay and decay components was determined by fitting this data with a one-decay-exponential and a constant convoluted with the Instrument Response Function (IRF) within a 0.7 ps timescale.

$$S_e(t) = IRF \otimes \left(a * e^{-\frac{t}{\tau_D}} + c \right)$$

Where 'a' and 'c' represent the amplitudes of the decay and non-decay components, respectively. 'τ_D' stands for the decay time constant, and 'IRF' denotes the instrumental response function. The population of the non-decay and decay components (NDC and DC) are calculated using the following equation:

NDP (%) =c/(a+c)

DP (%) =a/(a+c)

Supplementary Note 4. The discussion about hot hole transfer

We suppose that the transfer of hot holes may be more difficult than the transfer of hot electrons. This hypothesis was based on two reasons: (1) In general, for metallic material, electrons with higher mobility are the carriers. However, faster relaxation dynamics and lower mobility of hot holes make them harder to transfer when compared with hot electrons[6]; (2) As shown in Supplementary Fig. 26, the hot hole generated by a pump pulse, the hot hole would first transfer to the HOMO+2 state. Our TA spectra could probe the bleaching signal from the response between HOMO and LUMO. The transfer of generated hot holes (such as the response of transitions from HOMO+2 to LUMO, ~2.5 eV) may be not observed within the detection range (470-740 nm, 1.7-2.6 eV) of our experiment. But, at present, there is no certain evidence. The role of hot hole transfer is still further explored.

Supplementary Note 5. The discussion about resonant energy transfer.

We discuss the plasmon resonant energy transfer (i.e., the energy from the excited plasmon is transferred to the neutral excitation in the molecule) about the results of Fig. 3 g & h based on the polarization-dependent experiment. The polarized dependent TA experiment of MXene/MB shows almost isotropic for the MXene-contributed signals (500-600 nm in Supplementary Fig. 27a) while strong anisotropic for the STA-MB contributed signal (650-740 nm in Supplementary Fig. 27b). And the polarized dependent TA of MB (Supplementary Fig. 28a-c) show significantly anisotropic. If the STA-MB signals come from plasmon resonant energy transfer, the polarized dependent STA-MB signal should exhibit isotropic due to the isotropic signals of MXene. This result may indicate that the resonant energy transfer is not dominant.

Supplementary Note 6. Fitting analysis of STA-R6G dynamics.

In Supplementary Fig. 29, a long-time (2 ns) dynamic of STA-R6G with an 800 nm excitation is presented. Initially, we employed two exponential rise and two decay functions convoluted with an IRF to fit the data. The STA dynamics data could be well-fitted by two exponential rises (50±10 fs and 3.5±0.5 ps) and one decay (>2 ns). The slower growth and decay dynamics processes (3.5±0.5 ps and >2 ns) could be attributed

to lattice heat transfer and dissipation. The R6G is continually heated via PMHT because the molecules cover only a small portion of the MXene surface (Supplementary Note 1), and only a portion of the absorbed photon energy can be transferred to R6G through the NEIHT pathway. While most of the heat still exists in MXene that has not been covered by molecules. Therefore, the heat in this portion of MXene would be transferred to the molecules through the PMHT pathway. Subsequently, we focused on the dynamics occurring before heat diffusion (<10 ps). As a control, STA-MB with excitation at 800 nm (Supplementary Fig. 31a) and STA-R6G with excitation at 520 nm (Supplementary Fig. 31b) exhibited electron transfer and decay features within 1 ps. The two sets of dynamics data were effectively fitted by two growth and one decay component fitting. The first growth process indicates electron transfer from MXene to a molecule, while the decay process signifies the return of transferred electrons from the molecule to MXene. The second growth process indicated lattice heat transfer from MXene to molecules, as illustrated in Supplementary Fig. 31c. On the other hand, STA-R6G dynamics (Supplementary Fig. 31d) with excitation at 800 nm did not show a noticeable decay component. Although the dynamics data can be well-fitted by two rise and one decay component, it also fits well by only two growth components (see Supplementary Fig. 31e) without including the decay component. The dynamics feature of rapid growth without decay is consistent with heat transfer but inconsistent with electron transfer. To confirm that the fast growth within 100 fs is not derived from conventional phonon-meditated heat transfer (PMHT), we employed a single exponential growth of picosecond timescale to simulate the data. The simulation results indicated that the dynamics data do not agree with a single exponential rise, suggesting that the fast growth dynamics cannot be solely explained by conventional PMHT. Instead, it represents a novel heat transfer pathway with a time constant of 50 fs.

Supplementary references

1. Yang W, Liu Y, McBride JR, Lian T. Ultrafast and Long-Lived Transient Heating of Surface adsorbates on plasmonic semiconductor nanocrystals. *Nano Lett.* **21**, 453-461 (2021).
2. Losego MD, Grady ME, Sottos NR, Cahill DG, Braun PV. Effects of chemical bonding on heat transport across interfaces. *Nat. Mater.* **11**, 502-506 (2012).

3. Bonn M*, et al.* Phonon-versus electron-mediated desorption and oxidation of CO on Ru(0001). *Science* **285**, 1042-1045 (1999).
4. Tomko JA*, et al.* Long-lived modulation of plasmonic absorption by ballistic thermal injection. *Nat. Nanotechnol.* **16**, 47-51 (2021).
5. Ling Z*, et al.* Flexible and conductive MXene films and nanocomposites with high capacitance. *Proc. Natl. Acad. Sci. U. S. A.* **111**, 16676-16681 (2014).
6. Zhang Y, Guo W, Zhang Y, Wei WD. Plasmonic photoelectrochemistry: in view of hot carriers. *Adv. Mater.* **33**, 2006654 (2021).
